# Feeding, fecundity and lifespan in female *Drosophila melanogaster*

**DOI:** 10.1098/rspb.2008.0139

**Published:** 2008-04-22

**Authors:** Andrew I Barnes, Stuart Wigby, James M Boone, Linda Partridge, Tracey Chapman

**Affiliations:** 1 Research Department of Genetics, Evolution and Environment, University College LondonDarwin Building, Gower Street, London WC1E 6BT, UK; 2 School of Biological Sciences, University of East AngliaNorwich NR4 7TJ, UK

**Keywords:** mating costs, sex peptide, fruitless, ageing, seminal fluid, life history

## Abstract

Male seminal fluid proteins induce a profound remodelling of behavioural, physiological and gene signalling pathways in females of many taxa, and typically cause elevated egg production and decreased sexual receptivity. In *Drosophila melanogaster*, these effects can be mediated by an ejaculate ‘sex peptide’ (SP), which, in addition, contributes significantly to the cost of mating in females. Recent research has revealed that SP can stimulate female post-copulatory feeding, raising the possibility that the widespread female cost of mating could be due to over-feeding. In this study, we used *D. melanogaster* as a model to test this hypothesis. We first show that elevated post-mating feeding is dependent upon egg production and does not occur in sterile *ovo*^*D1*^ mutant females. This conclusion was also supported by the increase in feeding of virgin females whose egg production was experimentally elevated. We then demonstrated that sterile *ovo*^*D1*^ and fertile females experienced identical survival costs of mating, related to their frequency of mating and not to female feeding rate or to egg production. We conclude that female mating costs are not the result of over-feeding, but may be due to other, potentially more direct, effects of ejaculate molecules.

## 1. Introduction

The male seminal fluid proteins of invertebrates can profoundly remodel female behaviour, physiology and gene expression, and females of a wide range of taxa typically show increased egg production and decreased sexual receptivity following mating (e.g. Wolfner 1997, 2002; Chapman *et al*. 1998; Chapman 2001; Gillot 2003; Kubli 2003; Lawniczak & Begun 2004; McGraw *et al*. 2004). Although striking post-mating responses (PMRs) seem to be a universal feature of invertebrates, the identity of the seminal fluid molecules that cause them have been identified in only a few cases (Gillot 2003). The fruitfly *Drosophila melanogaster* has proven to be a valuable model system for the investigation of PMRs, and seminal fluid proteins are now known to cause a whole range of different PMRs including decreased receptivity, increased oogenesis, ovulation and antimicrobial peptide production, as well as playing central roles in processes such as sperm storage and retention, the processing of other seminal fluid proteins and in changes to the morphology of the reproductive tract (e.g. Herndon & Wolfner 1995; Tram & Wolfner 1998; Heifetz *et al*. 2000; Wolfner 2002; Bloch Qazi & Wolfner 2003; Chapman *et al*. 2003; Kubli 2003; Liu & Kubli 2003; Peng *et al*. 2005*b*; Ram *et al*. 2006; Adams & Wolfner 2007; Ram & Wolfner 2007*a*,*b*).

Recent attention has focused on a single remarkable ‘sex peptide’ (SP; Kubli 2003) that increases egg production (Chen *et al*. 1988; Soller *et al*. 1997, 1999; Chapman *et al*. 2003; Liu & Kubli 2003), causes the release of juvenile hormone (JH; Moshitzky *et al*. 1996); reduces female receptivity to further mating attempts (Chen *et al*. 1988; Chapman *et al*. 2003; Liu & Kubli 2003); stimulates the release of antimicrobial peptides (Peng *et al*. 2005*b*; Domanitskaya *et al*. 2007); stimulates female post-copulatory feeding (Carvalho *et al*. 2006); and reduces the fitness of females when they mate multiply (Chapman *et al*. 1995; Wigby & Chapman 2005). The latter involvement of SP in causing female mating costs is especially interesting, as such costs are taxonomically widespread (Bell & Koufopanou 1986; Partridge & Harvey 1988). Hence, understanding the mechanisms underlying such costs and establishing their generality is of fundamental importance for the evolution of life histories (Partridge & Harvey 1988). In this respect, the stimulation by SP of post-copulatory feeding in females (Carvalho *et al*. 2006), which presumably allows females to reproduce at an elevated level following receipt of sperm, is particularly intriguing. It suggests the hypothesis that mating costs in females may be caused by the deleterious effects of over-feeding (Carvalho *et al*. 2006), either as a direct effect or through increased fecundity. We test these hypotheses in this study.

Variation in feeding rate is itself an important life-history trait because it determines the level of nutrient intake (R. Wong, M. D. W. Piper and L. Partridge 2008, unpublished data), which in turn alters lifespan and reproductive rate (e.g. McCay *et al*. 1935; Weindruch & Walford 1988; Chapman & Partridge 1996; Partridge & Gems 2002; Partridge *et al*. 2005). For example, nutrient intake has a profound effect on lifespan across a wide range of species, with both very low and very high levels of dietary protein being associated with decreased longevity (e.g. Chapman & Partridge 1996; Partridge & Gems 2002; Partridge *et al*. 2005; Mair *et al*. 2005; Piper & Partridge 2007).

At a proximate level, exactly how the SP initiates its many different PMRs is mostly still unknown. Structure–function experiments (Schmidt *et al*. 1993; Kubli 2003) have revealed that, although only a short 36 amino acid peptide, SP appears to have different functional domains. The C-terminal end increases egg production and decreases receptivity and binds SP to its targets; the N-terminus causes the release of JH and binds to sperm (Kubli 2003) and the central region of SP may stimulate the female immune response (Domanitskaya *et al*. 2007). It is of interest to consider these mechanistic details, as they can help to reveal to what extent the full repertoire of SP phenotypes is linked and hence any probable constraints on their evolutionary trajectories. Even though SP is a single peptide, SP phenotypes could be free to evolve either because the cleavage of the peptide within the female (Peng *et al*. 2005*a*) separates functional domains, or because the same domain of SP acts upon different target tissues (Yapici *et al*. 2008) to produce different phenotypes. For example, egg production and receptivity PMRs were originally thought to be linked, but recent experiments reveal that the effect of SP on receptivity is independent of the presence of a mature egg in the uterus and the ability to produce and lay eggs (Barnes *et al*. 2007). Hence this study also initiates an investigation into the links between different SP phenotypes by revealing whether the effects of SP on female feeding are linked to increased egg production and/or to the female cost of mating.

We first tested whether sterile *ovo*^*D1*^ females (in which egg production arrests prior to vitellogenesis) and control fertile females show elevated feeding following SP transfer. We then examined the feeding rates of virgin females whose SP-like egg production and receptivity PMRs were activated by silencing *fruitless* (*fru*) neurons (Kvitsiani & Dickson 2006; Yapici *et al*. 2008). We then tested the hypothesis that the magnitude of female mating costs in fertile and sterile *ovo*^*D1*^ females is associated with female feeding rate and/or egg production.

## 2. Material and methods

### (a) Drosophila stocks

#### (i) Wild-type

The laboratory wild-type stock was collected in Dahomey (now Benin) in 1970 and has been maintained since then in large population cages with overlapping generations on a 12 L : 12 D cycle at 25°C. A *white*^Dahomey^ stock was generated by repeatedly backcrossing *w*^*1118*^ into the Dahomey genetic background (Broughton *et al*. 2005).

#### (ii) Sterile ovo^D1^ females

Flies with the *ovo*^*D1*^ mutant were obtained from the Bloomington stock centre (no. 1309) and the autosomes of this stock were then backcrossed at least nine times into *white*^Dahomey^. To produce sterile *ovo*^*D1*^ females for the first feeding experiment, males carrying the dominant *ovo*^*D1*^ mutation were crossed to *white*^Dahomey^ virgin females. Female offspring have ovaries that arrest and degenerate prior to S5, before vitellogenic uptake begins (Oliver *et al*. 1987). The *white*^Dahomey^ females were used as controls. In the main cost of mating experiment, sterile *ovo*^*D1*^ females were obtained by crossing *ovo*^*D1*^ males to virgin Dahomey females, with Dahomey females as controls. The Dahomey stock used was the one from which *white*^Dahomey^ was derived.

#### (iii) SP-lacking males

We obtained SP-lacking males by RNAi, as described in Chapman *et al*. (2003). The SP knockdown males were obtained by crossing males carrying a UAS-SP inverted repeat transgene (*UAS-SP-IR*) to females carrying an X-linked accessory gland-specific promoter (*Acp26Aa-P-Gal4*). The resulting SP knockdown males produced no detectable SP, as verified by Western blots (Chapman *et al*. 2003). Controls were obtained from the reciprocal cross of above, which controlled fully for autosomal genetic background.

#### (iv) Females with silenced fru neurons

Silencing of the neurons, which in males express the *fruitless* (*fru*) gene, leads to increased egg production and decreased sexual receptivity in virgin females, behaviours that are characteristic of SP-mediated PMRs (Kvitsiani & Dickson 2006). Silencing the transmission of *fru* neurons, therefore, provides a useful method to manipulate virgin females to behave as if mated, in terms of their egg production and receptivity. We silenced *fru* neurons in virgin females using a *UAS-shi*^*ts*^ transgene driven by *fru*^*GAL4*^, as in Kvitsiani & Dickson (2006). The *UAS-shi*^*ts*^ transgene was backcrossed into the Dahomey wild-type genetic background and the *fru*^*GAL4*^ was in a Canton-S genetic background. Experimental flies (*UAS-shi*^*ts*^/+; *fru*^*GAL4*^/+) were the female offspring of *UAS-shi*^*ts*^ females and *fru*^*GAL4*^ males. The two sets of ontrol flies were the offspring of *UAS-shi*^*ts*^ females mated to Canton-S males (i.e. *UAS-shi*^*ts*^/+), and the offspring of Dahomey females mated to *fru*^*GAL4*^ males (i.e. *fru*^*GAL4*^/+). Thus, all flies were the offspring of females with the Dahomey genetic background and males with the Canton-S genetic background. The experimental *UAS-shi*^*ts*^; *fru*^*GAL4*^ females show activation of SP-like egg laying and receptivity PMRs at the restrictive (29°C) but not permissive (18°C) temperature (Kvitsiani & Dickson 2006; S. Wigby 2007, unpublished data). The *UAS-shi*^*ts*^ and *fru*^*GAL4*^ stocks were kindly donated by Prof. Barry Dickson (Institute of Molecular Pathology, University of Vienna).

#### (v) poxn^70^, behaviourally sterile males

*poxn*^*70*^ was backcrossed eight times into the Dahomey background. In *poxn*^*70*^ null mutants, no chemosensory bristles form and although *poxn*^*70*^ males court normally, they cannot mate (Awasaki & Kimura 1997). *poxn*^*70*^/*CyO* flies were kindly donated by Dr Ken-ichi Kimura (Hokkaido University of Education).

### (b) Culturing conditions

All stocks were maintained and experiments run on standard sugar/yeast (SY) medium (100 g brewer's yeast powder, 100 g sugar, 20 g agar, 30 ml Nipagin (100 g l^−1^), 3 ml propionic acid and 1 l dH_2_O), supplemented with a drop of live yeast paste or with live yeast granules. To obtain experimental females, parental flies were reared on normal SY food before transfer to grape juice agar medium (50 g agar, 600 ml grape juice (Continental Wine Experts Limited; Cawston, Norwich), 42 ml Nipagin (100 g l^−1^) and 1 l dH_2_O) for oviposition and collection of eggs for standard density cultures, as described in Clancy & Kennington (2001). Males were grown in standard or low-density cultures.

### (c) Effect of SP receipt and female sterility on feeding rate

We conducted behavioural assays of female feeding rates as opposed to assays based on the uptake of radioisotopes or dyes (Carvalho *et al*. 2005, 2006) because the latter methods can run into potential problems resulting from experimental treatments altering the fly's capacity for the label, thus differentially altering the retention time of that label (Wong *et al*. 2008). Direct behavioural observation of proboscis extension therefore offers a more accurate alternative measure of steady-state feeding rate (Mair *et al*. 2005; Wong *et al*. 2008), and it is tightly correlated with food consumption (R. Wong, M. D. W. Piper and L. Partridge 2008, unpublished data). To ensure that our behavioural measure gave the same result as previously reported (Carvalho *et al*. 2006) and to test whether the effect of SP on feeding is observed in sterile *ovo*^*D1*^ females, we measured the feeding rates of *ovo*^*D1*^ females and controls mated to SP knockdown or control males. We placed sexually mature fertile wild-type and sterile *ovo*^*D1*^ virgin females with either two SP-transferring or SP-lacking males each. There were 20 females per group and after mating the males were removed. Feeding assays were performed in the afternoon, 2–4 hours after mating. For all feeding assays in this study, the observer entered the room and waited for 15 min before starting the assay, to allow flies to settle after any disturbance caused, and in all experiments the vials were coded blind. Each vial was scanned 20 times during the 2 hour period of the assay. Feeding was scored if a female fly displayed an extended proboscis onto the food surface and was recorded as the number of females observed to feed during each set of observations.

### (d) Effect of silencing *fruitless* neurons on female feeding rate

We tested whether the activation of SP-like egg laying and receptivity PMRs was sufficient to increase feeding rate in virgin females. We used *UAS-shi*^*ts*^ and *fru*^*GAL4*^ flies to inactivate *fru* neurons in females as described in Kvitsiani & Dickson (2006). The females were grown at standard density at 18°C, collected as virgins within 16 hours of eclosion (day 0), placed five per vial and maintained at 18°C. At 5 days post-eclosion, half the vials of each genotype were allocated randomly to the restrictive (29°C) or permissive (18°C) temperature treatments. The next day (day 6), all flies were transferred onto fresh food. The 29°C feeding assay was performed on day 7 and the 18°C assay 1 day later. Both assays were performed approximately 1.5 hours after lights on. Sixteen vials per experimental group (each containing five females) were transferred to observation racks the night before the assay. Vials were scanned continuously and female feeding behaviour was scored until all vials had been observed at least 15 times. Feeding was scored as described above and was recorded as the number of females observed to feed in each vial during each observation.

### (e) Mating costs and feeding rates in fertile and sterile *ovo*^*D1*^ females

To test for costs of mating, sterile *ovo*^*D1*^ and fertile Dahomey females were subjected to ‘high’ and ‘low’ cost of mating treatments (i.e. continuous or intermittent exposure to wild-type males; Fowler & Partridge 1989), while controlling for non-mating costs of exposure to males in the low-cost conditions by using behaviourally sterile *poxn*^*70*^ males. Virgin females were collected over ice in a single 24 hour time period, and then housed in groups of three. Females were continuously housed with males in a 1 : 1 sex ratio for the duration of the experiment. The ‘low-cost’ groups were housed with fertile wild-type males for 24 hours, then lightly anaesthetized with CO_2_ and the wild-type males replaced with non-mating (*poxn*^*70*^) males. These males were kept with the females for 72 hours, after which they were replaced with wild-type males once again. Thus, the males were cycled in this manner every 4 days until all females were dead. The ‘high-cost’ groups were subjected to a similar cycle, except that wild-type males were transferred in on days 2–4, rather than non-mating *poxn*^*70*^ males. Males were not re-used during the experiment, but were replaced with younger males not more than 4 days old at the beginning of each 4-day cycle. All males for these replacements were obtained from stock bottles maintained at similar densities. Therefore, high-cost groups were exposed to mating males continuously throughout their lifetimes, whereas low-cost groups received continuous courtship, but were exposed to mating males for only 1 day out of every 4-day cycle. Female mortality was scored daily and 70 vials per experimental group were assayed, giving a total of 210 females per group.

Feeding assays were performed on subsets of vials from the longevity experiment. Assays were begun shortly after lights on (at 10.00) and lasted for approximately 1.5 hours. Twenty vials per experimental group (each containing three males and three females, as above) were transferred to observation racks the night before the assay. Vials were scanned continuously and female feeding behaviour was scored until all vials had been observed at least 10 times. Feeding was scored as described above and recorded as the number of females observed to feed in each vial during each observation. Feeding was assayed twice during the 4-day cycle: once on the morning of day 2 prior to male transfer, when all females were with wild-type males, and once on the morning of day 4, when females were with wild-type or *poxn*^*70*^ males. Assays were performed on days 2, 6 and 10 (all females with wild-type males) and days 4, 8 and 12 (females with wild-type or *poxn*^*70*^ males). Vials that contained fewer than three females owing to previous mortality were not included in the assays.

To ensure that the manipulations had the desired effect of varying mating frequency while keeping any non-mating costs of exposure to males constant, we monitored mating frequency and courtship throughout the experiment. Male courtship was sampled on a subset of 20 vials per experimental group from the longevity experiment and was assayed at similar times and under similar conditions as the feeding assays described above. We similarly recorded mating frequency for the days on which females were paired with wild-type males. Vials were scanned continuously and mating or courting behaviour was scored until all vials had been observed at least 10 times. Courting was scored for each male that showed one of a number of typical courtship behaviours (orientation towards the female, wing extension at 90°, trailing females, genital contact with proboscis and attempting to mount the female). Courtship was recorded as the number of courting males in each vial during each observation. The vials were again coded blind. Male courtship was assayed once during each 4-day cycle: on day 3 when females with either wild-type or *poxn*^*70*^ males. Assays were performed on days 3, 7, 11 and 15 of the longevity screen.

To check that survival patterns in the fertile females were indicative of costs and were not confounded by differences in age-specific fecundity schedules, we recorded fecundity for a subset of 20 vials per experimental group (excluding the sterile *ovo*^*D1*^ females) for eggs laid between days 1 and 2 of the 4-day cycle, when all females were housed with wild-type males. The males were transferred into these vials starting at 14.00 and were transferred again at 12.00 the following day, so that each vial contained the three females and wild-type males for exactly 22 hours. After the flies had been transferred, vials were retained and egg counts performed on days 6, 10, 14 and 18 of the longevity screen. A bacterial infection in some vials reduced the sample sizes slightly: low-cost controls: day 6=15 vials, day 10=7 vials, day 14=18 vials, day 18=22 vials; high-cost controls: day 6=14 vials, day 10=10 vials, day 14=16 vials, day 18=17 vials. On day 6, we measured egg–adult viability by retaining vials after egg counting, to record the number of adults that emerged 10–12 days later.

### (f) Statistical analysis

Feeding data were analysed using ANOVAs, and *post hoc* multiple comparisons between treatments were made using Tukey's HSD tests. For some tests, Box–Cox transformations were used to improve the normality of the data. In the cost of mating experiment, multiple samples were taken from the same population (i.e. for feeding, fecundity, courtship and mating measures). Therefore, to avoid the problems associated with pseudoreplication we analysed feeding, fecundity and courtship data using repeated measures ANOVAs. Mating rates were analysed by comparing the total number of observed mating opportunities taken and not taken across the whole experiment, using Pearson's chi-squared tests. We corrected for the fact that the low-cost groups were housed with non-mating males for 3 out of every 4 days. Thus, in the low-cost groups, the ‘number of mating opportunities taken’ was equal to the number of females observed mating during the assays, but the ‘number of mating opportunities not taken’ was the number of non-mating females observed during the assays, plus three times the total number of observed females (because none of these females had the opportunity to mate for the 3 days when they were with non-mating males). Survival data were analysed using proportional hazards. All analyses and transformations were performed using JMP (v. 5.1, 1989–2003 SAS Institute, Inc.) except for repeated measures ANOVAs that were performed using SPSS (v. 13, SPSS, Inc., 1989–2006). Accurate *p* values from Tukey's HSD tests were obtained using R (Ihaka & Gentleman 1996).

## 3. Results

### (a) Effect of SP receipt and female sterility on feeding rate

**Figure 1:**
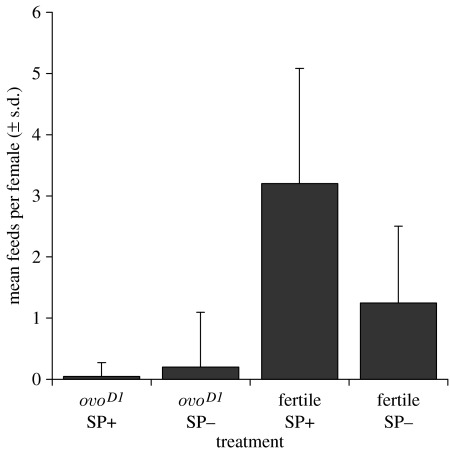
Feeding rates (mean feeds per female±s.d.) of control and sterile *ovo*^*D1*^ females mated to SP-transferring and SP-lacking males.

The feeding data were first Box–Cox transformed to improve normality. As expected, SP transfer caused a significant increase in the feeding rate of fertile females (figure 1). There were significant effects due to SP (*F*_1,76_=6.421, *p*=0.0133) and female genotype (*F*_1,76_=113.47, *p*<0.0001) and their interaction (*F*_1,76_=7.723, *p*=0.0069). Fertile females fed significantly more than sterile *ovo*^*D1*^ females (*q*_76,4_>7.874, *p*<0.0001 for all comparisons), and receipt of SP-stimulated feeding in fertile females (*q*_76,4_=5.313, *p*=0.0018) but not sterile *ovo*^*D1*^ females (*q*_76,4_=0.245, *p*=0.998). Our behavioural measure therefore detected SP-mediated differences in feeding rate in fertile females, as reported for alternative methods (Carvalho *et al*. 2006). Our results show that egg production is required for the effect of SP on female feeding to be observed.

### (b) Effect of silencing *fru* neurons on female feeding rate

**Figure 2:**
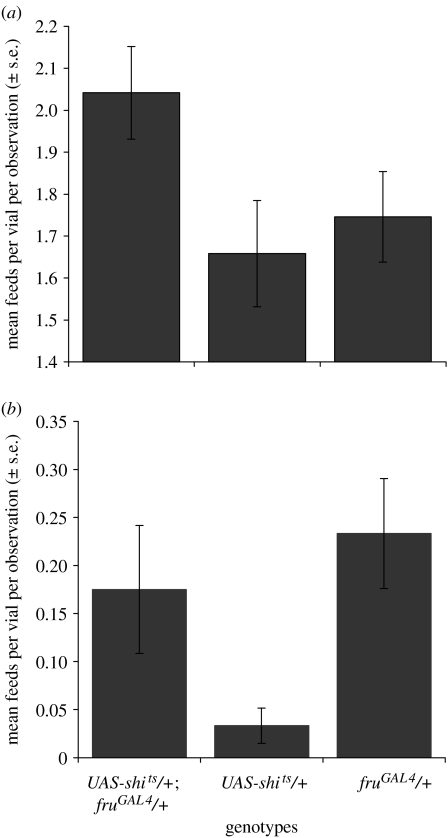
Feeding rates in virgin females with silenced *fru* neurons. Feeding rates (mean per vial per observation±s.e.) for experimental (*UAS-shi*^*ts*^/+; *fru*^*GAL4*^/+) and control (*UAS-shi*^*ts*^/+ or *fru*^*GAL4*^/+) virgin females at (*a*) restrictive (29°C) and (*b*) permissive (18°C) temperatures.

To test whether the silencing of *fru* neurons, which activates SP-like receptivity and egg-laying PMRs (Kvitsiani & Dickson 2006), is sufficient to increase feeding rate, we analysed the number of feeds per vial in a nested ANOVA with ‘experimental treatment’ (i.e. *fru* silenced versus control flies) and ‘genotype’ nested within experimental treatment as factors. The data were analysed separately for 18 and 29°C, as it was not possible to conduct simultaneous behavioural observations at both temperatures. The data from the 18°C experiment were Box–Cox transformed to improve normality. Feeding rates were much higher at 29°C than at 18°C (figure 2). At the restrictive temperature (29°C), the females with silenced *fru* neurons fed at a significantly higher frequency than controls (*F*_1,45_=5.78, *p*=0.02; figure 2*a*) and there was no significant effect of control genotype on feeding rate (*F*_1,45_=0.28, *p*=0.59). These results were consistent with the finding that other SP-like oviposition and receptivity PMRs are also activated in females in which *fru* neurons are silenced (Kvitsiani & Dickson 2006). At the permissive temperature there was no difference between the experimental and control females in feeding rate (*F*_1,45_=0.00, *p*=0.99; figure 2*b*) but there were differences between genotypes (*F*_1,45_=10.42, *p*=0.002). Although there was a significant difference in feeding rate between the two control female treatments (*fru*^*GAL4*^/+ control females fed significantly more than *UAS-shi*^*ts*^/+ control females; *q*_45,3_=4.56, *p*=0.007), there were no significant differences between the experimental (*UAS-shi*^*ts*^/+; *fru*^*GAL4*^/+) versus either of the control groups of females (*q*_45,3_<0.283, *p*>0.25 for both comparisons). It is unclear why the two control genotypes differed from one another in feeding rate at the permissive temperature; however, the 18°C results show that the increase in feeding rate of the *fru* silenced females seen at the restrictive temperature is not due to an effect of genetic background or position effects of transgenes used. Our results demonstrate that the experimental elevation of egg laying in virgin females by the silencing of *fru* neurons is associated with elevated feeding. This supports the finding from the first experiment that egg production is necessary for increased feeding following mating.

### (c) Mating costs and feeding rates in fertile and sterile *ovo*^*D1*^ females

We first analysed the mating and courtship data to ensure that our high- and low-cost treatments did indeed have the desired effect of varying female mating frequency, while controlling for the effects of non-mating exposure to males. As expected, the high-cost groups mated significantly more in their lifetime than did the low-cost groups (table 1). This was true for both the fertile (*Χ*_1_^2^=17.11, *p*<0.0001) and sterile *ovo*^*D1*^ (*Χ*_1_^2^=8.34, *p*=0.004) females. The number of matings did not differ between fertile or sterile *ovo*^*D1*^ females within high- and low-cost treatments (high-cost *Χ*_1_^2^=0.66, *p*=0.417; low-cost *Χ*_1_^2^=0.04, *p*=0.840). There were no significant differences in the amount of courtship observed in the different treatments during the experiment, and hence, given that courtship was assayed on days when low-cost groups were exposed to *poxn*^*70*^ males, there was no difference in the amount of courtship delivered by *poxn*^*70*^ versus wild-type males (mean courts/vial/observation/day for fertile females, high cost=0.623, low cost=0.600; and sterile *ovo*^*D1*^ females, high cost=0.554, low cost=0.607; *F*_3,9_=0.327, *p*=0.806). The data show that the high-cost treatments received significantly more matings than low-cost treatments, and that this effect occurred similarly for both sterile *ovo*^*D1*^ and fertile females. Furthermore, we detected no differences in the non-mating costs of exposure to males across the groups, with wild-type and *poxn*^*70*^ males courting females at comparable rates.

**Figure 3:**
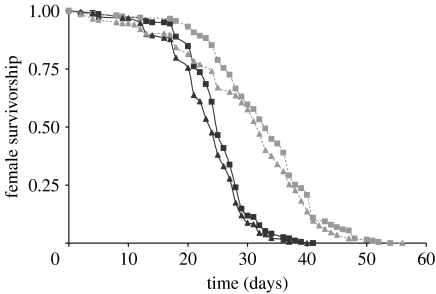
Survivorship against time in days for control and sterile *ovo*^*D1*^ females exposed to high and low cost mating regimes (continuous versus intermittent exposure to mating males, respectively). (Black squares and triangles, high-cost sterile *ovo*^*D1*^ and fertile females respectively; grey squares and triangles, low-cost sterile *ovo*^*D1*^ and fertile females, respectively.)

The low-cost mating groups lived significantly longer than the high-cost mating groups in both the control and the sterile *ovo*^*D1*^ females (figure 3). Mating treatment (low or high cost) had a significant effect on survival (likelihood ratio *Χ*_1_^2^=198.95, *p*<0.0001; figure 3), as did female genotype (*Χ*_1_^2^=7.12, *p*=0.0076). However, there was no significant interaction between the two effects (*Χ*_1_^2^=0.03, *p*=0.85). Comparisons within treatments show that low-cost mating groups lived significantly longer than the high-cost mating groups in both the fertile and the sterile *ovo*^*D1*^ females (*Χ*_1_^2^>90.1, *p*<0.0001 for both comparisons). Sterile *ovo*^*D1*^ females were longer lived than fertile females in the high-cost treatment, and marginally non-significantly longer lived in the low-cost treatment (high cost: *ovo*^*D1*^ median lifespan=25 days, fertile median lifespan=24 days, *Χ*_1_^2^=4.18, *p*=0.041 and low cost: *ovo*^*D1*^ median lifespan=33 days, fertile median lifespan=32 days, *Χ*_1_^2^=3.08, *p*=0.079). Overall, the results show that survival costs of mating were incurred to an equal degree by both sterile *ovo*^*D1*^ and fertile females.

**Figure 4:**
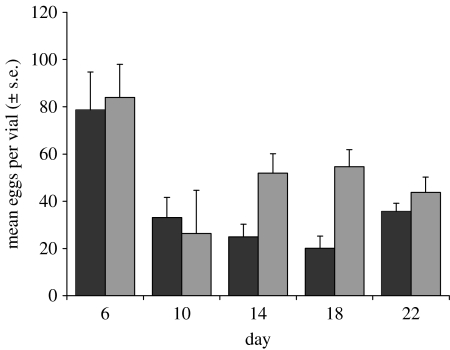
Mean (±s.e.) egg laying rate against time in days for control females exposed to the fertile high-cost (black bars) and low-cost (grey bars) mating regimes as shown in figure 3.

The survival results among the fertile females could be confounded by differences in fecundity, if, for example, high-cost females had shorter lifespans but higher age-specific fecundity. However, analysis of the mean fecundity data per day showed no evidence for this and indeed a non-significant trend for the opposite effect, i.e. for low-cost females to lay more eggs than high-cost females (*F*_1,4_=3.264, *p*=0.145; figure 4). The egg-to-adult viability data obtained on day 6 showed no significant differences between the low- and high-cost groups (*F*_1,44_=0.0008, *p*=0.978). Taken together, the fecundity and fertility results show that the reduced survival suffered by high-cost females was not a result of any changes in the fecundity or fertility schedule.

**Figure 5:**
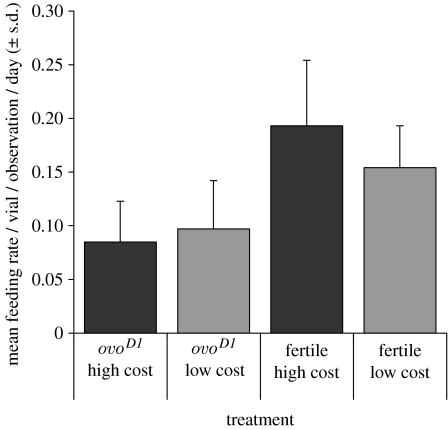
Mean (±s.d.) female feeding rates/vial/observation/day for control and sterile *ovo*^*D1*^ females exposed to the high- and low-cost mating regimes as shown in figure 3. Females exposed to either non-mating or wild-type males did not differ significantly in feeding rate (see text) and the data for all feeding assays were therefore combined.

We analysed feeding rates to ask whether any differences between treatments mapped onto the survival patterns, as would be expected if feeding were associated with increased mating costs. First, we tested whether male genotype (*poxn*^*70*^ or wild-type) affected female feeding rate. We found no significant difference in feeding rates on days in which females were exposed to *poxn*^*70*^ versus wild-type males. This was true for both fertile females (*F*_1,3_=2.33, *p*=0.224) and sterile *ovo*^*D1*^ females (*F*_1,3_=1.15, *p*=0.361; figure 5). Hence females exposed to *poxn*^*70*^ versus wild-type males fed at equivalent rates, and these data were combined in the main analysis. However, it should be noted that analysis of the data using only the days on which all females were exposed to wild-type males gives equivalent results to those described below (data not shown). There were significant differences in feeding rates between treatments (*F*_3,21_=9.355, *p*<0.001). Fertile females fed more than sterile *ovo*^*D1*^ females. High-cost fertile females fed significantly more than both high- and low-cost sterile *ovo*^*D1*^ females (high-cost fertile versus high-cost *ovo*^*D1*^, *q*_21,6_=6.85, *p*=0.001 and high-cost fertile versus low-cost *ovo*^*D1*^, *q*_21,6_=6.07, *p*=0.004). This effect was less marked for the fertile low-cost treatment (low-cost fertile versus high-cost *ovo*^*D1*^, *q*_21,6_=4.38, *p*=0.053 and low-cost fertile versus low-cost *ovo*^*D1*^, *q*_21,6_=3.61, *p*=0.153; figure 5). However, we detected no significant differences between high- versus low-cost fertile females (*q*_21,6_=2.46, *p*=0.522) or between high- versus low-cost sterile *ovo*^*D1*^ females (*q*_21,6_=0.77, *p*=0.99). Taken together with the survival analyses, the results indicate that feeding rate (high in fertile females and low in sterile *ovo*^*D1*^ females) was not associated with the magnitude of mating cost experienced (high in fertile and sterile *ovo*^*D1*^ high-cost treatments and low in fertile and sterile *ovo*^*D1*^ low-cost treatments). Female feeding rate was instead associated with the ability to produce and lay eggs.

## 4. Discussion

In this study we set out to test, using the SP ejaculate peptide of *D. melanogaster* as a model, whether the elevated feeding that is seen in females following mating is a potential explanation for the widespread cost of mating in females across many taxa, via the deleterious effects of overeating. Our results clearly do not support this hypothesis, as they show that the extent of the female survival cost of mating was associated with the frequency of mating and not with female feeding. The lack of association between egg production and survival costs of mating is consistent with previous studies (Chapman *et al*. 1995; Chapman & Partridge 1996) and that lifespan and fecundity do not show an obligate trade-off (e.g. Hwangbo *et al*. 2004; Mair *et al*. 2004; Barnes *et al*. 2006). Our results highlight new mechanistic details of the SP-mediated elevated feeding seen in females after mating in showing that this effect is dependent on the ability to produce and lay eggs. This conclusion is also supported by the data from the experiments in which we detected increased feeding rates in virgin females in which egg laying was experimentally elevated by silencing *fru* neurons (Kvitsiani & Dickson 2006). The results also shed light on the associations between the many different PMRs and show that they are sometimes (e.g. feeding and egg production/laying, this study) but not always (e.g. receptivity and egg production/laying; Barnes *et al*. 2007) linked.

Generally, the results do not provide support for the idea that increased feeding can explain a major part of reproductive costs in females, and so alternative explanations still need to be sought. For *D. melanogaster* this may include more direct and perhaps toxic side effects of ejaculate molecules whose primary function is to increase male reproductive success (Chapman *et al*. 1995; Wigby & Chapman 2005). Further general implications are that increases in female feeding following mating to fuel the increased demands of higher reproductive rates (and which have not yet been widely tested for, but which are predicted to be common) may sometimes be accompanied by changes in other life-history traits such as egg production. The existence, or not, of links between PMRs caused by the same effector (SP) is interesting in terms of how coordinated sets of PMRs actually evolve. Presumably, evolution is less constrained if the phenotypes under selection are not strongly linked. Molecules like SP, which can apparently control so many different PMRs, therefore offer a fascinating opportunity to investigate both theoretically and empirically, the process of recruitment and evolution of new PMRs.

Turning to the results in more detail, the cost of mating experiment revealed that fertile and sterile *ovo*^*D1*^ females suffered equivalent survival mating costs, in the absence of any confounding differences in non-mating exposure to males or differences in fecundity among the fertile females. Female feeding rate was significantly higher in fertile than in sterile *ovo*^*D1*^ females, again supporting the association between feeding and the ability to produce and lay eggs. However, the major result from this experiment was that the magnitude of mating costs was not related to female feeding rate. Mating costs were instead associated with female mating frequency. Within high- and low-cost treatments, the survival of sterile *ovo*^*D1*^ females was marginally higher than for fertile females, which might indicate a slight survival benefit of feeding less, but this is not borne out by the much larger difference in survival between high- and low-cost treatments for fertile and sterile *ovo*^*D1*^ females, which occurred independently of differences in feeding rates. We conclude that the survival cost of mating in females cannot be explained by SP-mediated alterations in female feeding rates, and that it also occurs over and above any costs of egg production. This argues that the processes that shorten lifespan when nutritional intake is elevated (Chapman & Partridge 1996; Partridge *et al*. 2005; Piper & Partridge 2007) may not be the same as those that cause the female survival cost of mating. The ejaculate-mediated cost of mating therefore may be due to other effects, such as direct seminal fluid toxicity (Mueller *et al*. 2007) or stimulation of the immune system (Peng *et al*. 2005*b*; Domanitskaya *et al*. 2007).

The presence or absence of links between the different processes affected by SP, in combination with knowledge about the targets of SP (Yapici *et al*. 2008), may help to initiate a proximate study of the different molecular pathways involved. For example, recent research shows that the sex peptide receptor (SPR) is expressed in a subset of *fru* neurons, as well as in other sites in the nervous system and in the female reproductive tract (Yapici *et al*. 2008). Receptivity and egg-laying PMRs can be significantly induced by SP in females that express SPR only in the *fru* neurons, which is consistent with the hypothesis that SP triggers these PMRs primarily by modulating the activity of the SPR-expressing *fru* neurons (Yapici *et al*. 2008). The effect of SP on receptivity occurs equally in both sterile *ovo*^*D1*^ and fertile females (Barnes *et al*. 2007), but the effect of SP on feeding does not occur in sterile *ovo*^*D1*^ females (this study). Feeding could therefore be controlled by a different molecular pathway to that which regulates receptivity and egg production/laying PMRs (such as the proposed SP/SPR/*fru* pathway), or by the same pathways, but with excitatory/inhibitory inputs signalled by the current rate of egg production/laying. The nature of the phenotypes controlled by the other SPR-expressing tissues (e.g. other sites in the nervous system and in the female reproductive system; Yapici *et al*. 2008) are topics of interest for future study.

## Figures and Tables

**Table 1:** Mating frequencies of fertile and sterile *ovo*^*D1*^ females exposed to high- and low-mating regimes.

female	cost of mating	mating opportunities taken	mating opportunities not taken	mating (%)
fertile	high	15	195	7.14
	low	12	828	1.43
*ovo*^*D1*^	high	11	199	5.24
	low	13	827	1.57
